# Biosynthesis of Inorganic Nanoparticles: A Fresh Look at the Control of Shape, Size and Composition

**DOI:** 10.3390/bioengineering4010014

**Published:** 2017-02-18

**Authors:** Si Amar Dahoumane, Clayton Jeffryes, Mourad Mechouet, Spiros N. Agathos

**Affiliations:** 1 School of Biological Sciences & Engineering, Yachay Tech University, Hacienda San José s/n, San Miguel de Urcuquí 100119, Ecuador; spiros.agathos@uclouvain.be; 2 Nanobiomaterials and Bioprocessing (NAB) Laboratory, Dan F. Smith Department of Chemical Engineering, Lamar University, P.O. Box 10053, Beaumont, TX 77710, USA; cjeffryes@lamar.edu; 3 Laboratoire de Physique et Chimie des Matériaux, Université Mouloud Mammeri, Route de Hasnaoua, BP 17 RP, Tizi-Ouzou 15000, Algeria; mechouetmourad@yahoo.fr; 4 Laboratory of Bioengineering, Earth and Life Institute, Université Catholique de Louvain, Croix du Sud 2, Bte L7.05.19, B-1348 Louvain-la-Neuve, Belgium

**Keywords:** biosynthesis, nanoparticles, screening, size, shape, composition, properties, bio-applications

## Abstract

Several methodologies have been devised for the design of nanomaterials. The “Holy Grail” for materials scientists is the cost-effective, eco-friendly synthesis of nanomaterials with controlled sizes, shapes and compositions, as these features confer to the as-produced nanocrystals unique properties making them appropriate candidates for valuable bio-applications. The present review summarizes published data regarding the production of nanomaterials with special features via sustainable methodologies based on the utilization of natural bioresources. The richness of the latter, the diversity of the routes adopted and the tuned experimental parameters have led to the fabrication of nanomaterials belonging to different chemical families with appropriate compositions and displaying interesting sizes and shapes. It is expected that these outstanding findings will encourage researchers and attract newcomers to continue and extend the exploration of possibilities offered by nature and the design of innovative and safer methodologies towards the synthesis of unique nanomaterials, possessing desired features and exhibiting valuable properties that can be exploited in a profusion of fields.

## 1. Introduction

The control of the shape, size and composition of inorganic nanomaterials is of paramount importance as these features provide them with attractive and often unique properties that can find applications in different fields, such as biomedicine [1], catalysis [2] and sensing [3]. While spherical nanogold displays a unique surface plasmon resonance (SPR) band, gold nanorods possess two SPR bands, the transverse and the longitudinal modes [4]. The latter red-shifts when the aspect-ratio, i.e., the ratio between the length and the width, increases. This property is exploited in cancer cell imaging and hyperthermia photo-treatment [5] as the longitudinal mode can be tuned to fit within the biological window of water [6]. Likewise, the shape of silver nanoparticles (Ag NPs) impacts dramatically their catalytic properties. For instance, cubic Ag NPs offer higher yields in the oxidation of styrene [7]. Similarly, the magnetic properties of cobalt oxide nanoparticles are influenced by their shapes and sizes [8].

To obtain a nanomaterial with desired shape and size, materials scientists can tailor the experimental sets. For instance, via the polyol process, it is possible to orient the synthesis of zinc oxide (ZnO) NPs towards obtaining different shapes and sizes by the right choice of the experimental parameters, such as the hydrolysis ratio (n(H_2_O)/n(Zn^2+^)) and the surfactant used [9]. Through the same process, other parameters, such as the salt precursor, the concentration of sodium hydroxide ([NaOH]) and the temperature ramping rate, impact the features of cobalt nanorods [10]. Similar procedures are implemented, for instance, to direct the shape and control the size of metallic nanomaterials [11].

The last two decades have witnessed the groundbreaking advent of the biosynthesis of inorganic nanoparticles which may be defined as the utilization of biological resources, such as biomolecules extracted from plants, bacteria or fungi, whole cells or parts of cells, to promote the production of nanomaterials starting from aqueous solutions of the corresponding salts. This field is growing fast and diversifying tremendously into a number of fascinating sub-disciplines. Generally, these procedures are implemented in aqueous media, usually at room temperature or under mild heating, and at atmospheric pressure. Moreover, the metallic salts are used as received and there is no need for further synthesis. These sustainable experimental practices, based on the exploitation of natural resources, fit within the criteria of Green Chemistry [12]. Several reviews depict to various degrees the procedures followed and the nanomaterials synthesized through the use of biological entities like bacteria [13,14], extremophiles [15], fungi [16,17,18], yeast [19,20], plants [21,22], algae [23,24,25,26], and natural biomolecules [27,28], or via the exploitation of templates based on cell constituents [29]. Although it is not their main scope, a few reviews highlight the shape and size of biogenic nanomaterials. For instance, the features of Au NPs synthesized via fungal-mediated routes were outlined by Kitching et al. [30] whereas those produced through plant-mediated methods were summed up by Noruzi [31]. The bio-applications of these biogenic nanocrystals are described, to varying extents, within the above cited reviews or extensively summed up in the review by Schröfel et al. (2014) [32].

The aim of the present review is to provide the readership, for the first time, with a more specific focus upon the control of the size, shape and composition of biogenic inorganic nanomaterials obtained through processes relying on renewable and sustainable resources. Our review encompasses the diverse biological resources screened, so far, for their biocatalytic capabilities in the fields of nanoscience and nanotechnology via several methodologies. As a result, the diversity of bioresources and methods of their preparation and exploitation ensure a wide variety in the produced nanocrystals, such as those made of noble metals, carbonates, oxides and chalcogenides, which, in turn, may display a formidable array of shapes, sizes and compositions, by tuning experimental parameters. To illustrate this fact, a few comprehensive examples, picked from the literature, are detailed. Furthermore, we describe in detail, when available, the mechanistic aspects that govern the generation of the NPs with desirable features and valuable properties. Finally, the selected findings are summed up and possible future developments are addressed.

## 2. Control of Size and Shape of Biologically Synthesized Metallic Nanoparticles

### 2.1. Gold

**Figure 1:**
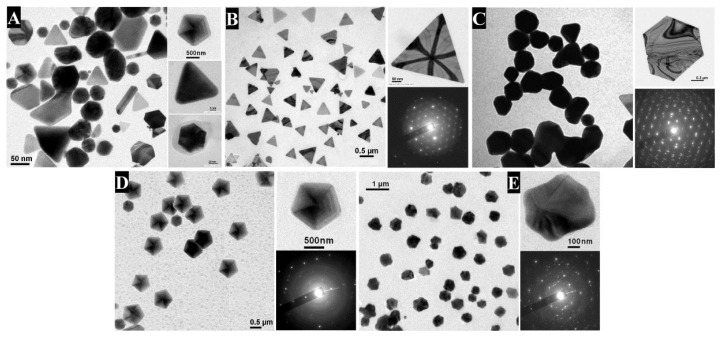
TEM images of Au NPs synthesized by mixing the cell-free aqueous extract of the fungus *Rhizopus oryzae*, obtained by tuning the experimental parameters: the amount of HAuCl_4_, the pH and the reaction time. (**A**) Mixed nanoplates (triangle, hexagon, pentagon, star, etc.). Right-hand images display individual nanoplates. Scale bars, clockwise from left are 50, 500, 5 and 20 nm. (**B**) Triangular nanoplates. Scale bars, clockwise from left are 500 and 50 nm. (**C**) Hexagonal nanoplates. No scale bars were given in the original publication except for 200 nm for the top right figure. (**D**) Pentagonal nanoplates. All scale bars are 500 nm. (**E**) Star-shaped nanoplates. Scale bars clockwise from left are 1 µm and 100 nm. For **B**, **C**, **D** and **E**, the upper picture of each right-hand image corresponds to the high-resolution single-crystalline nanoplates, and the lower image to their corresponding selected area electron diffraction (SAED) patterns, respectively. Reproduced from Reference [36] with permission from Wiley.

Several biological resources have proven efficient in carrying out the reduction of cationic gold into its metallic counterpart and, thereby, the production of Au NPs [33]. Among these resources, the cell-free extract of *Rhodopseudomonas capsulata* promotes the production of, either, spherical Au NPs or Au nanowires when the concentration of auric cations is doubled for the same amount of the extract through the aggregation of firstly formed spherical Au NPs and their oriented growth towards the appearance of Au nanowires [34]. The extract of the edible mushroom *Volvariella volvacea*, once in contact with auric cations, promotes the production of Au NPs whose shape and size depend on the volume of fungal biomass extract used [35]. In fact, by varying the content of the extract in the reaction medium, it is possible to obtain different shapes for the NPs, as illustrated by transmission electron microscopy (TEM) images. For instance, at the lower extract content, spheres and triangles are obtained, while, at the higher extract content, flower-like NPs are produced. Using the cell-free aqueous extract of the fungus *Rhizopus oryzae*, Das et al. detailed the production of Au NPs under different shapes by tuning the experimental parameters, namely the reaction time, the concentration of HAuCl_4_ and the pH (Figure 1) [36]. Specifically, triangles are produced when 1000 mg/L of Au(III) are used, the pH is adjusted to 8 and the reaction lasts 10 h. Hexagons are obtained when 1500 mg/L of Au(III) are used, the pH is adjusted to 3 and the reaction lasts 10 h. Interestingly, it is possible to favor one shape at the expense of another by just a slight tuning of the abovementioned parameters. For instance, longer reaction time for the experiment that yielded gold wires will promote the production of gold rods. On the other hand, switching the pH from 6 to 8 and for an extra 4 h of reaction, star-shaped Au NPs are obtained instead of pentagons. In all reactions, the cell-free aqueous extract of *Rhizopus oryzae* acts as the pool of reducing and stabilizing agents. Although the mechanism was not biochemically elucidated, there is no doubt as to the action of proteins contained within the fungal extract in the process of reduction of Au(III) to Au(0) and the subsequent formation of shape-directed Au NPs (see below).

In a similar way, numerous studies have reported on the ability of plant extracts to direct the shape and the size of Au NPs. Working with the right experimental sets, Arockiya Aarthi Rajathi et al. reported the synthesis of spherical Au NPs of ~13 nm in diameter using leaf biomass of *Suaeda monoica* [37]. Kasthuri et al. described the production of gold nanoplates using apiin extracted from Henna [38]. The increase of the apiin affects the shape and the size of the as-produced Au NPs: at the lower content, nanoplates are mainly obtained; at the intermediate content, less nanoplates and more round-shaped nanoparticles are observed; and, at the higher content, only round-shaped nanoparticles are garnered. Another pure active ingredient, caffeine, is used to fashion gold nanowires [39]. The extract of *Aloe vera* leads to a mixture of Au NPs in the shape of triangles and spheres in the presence of HAuCl_4_ [40]. To shed light on the biochemical pathways governing the production of Au NPs of different shapes, proteins contained in the extract of *A. vera* were separated using a 3 kDa cutoff dialysis membrane. It was found that only Fraction 1, with a molecular weight of less than 3 kDa, was able to promote the reduction of cationic gold and, significantly, yield the production of hexagonal and triangular nanoplates. These primary findings were corroborated by the results obtained by Xie et al. who, soon after, carried out a more detailed work in order to elucidate the mechanism involved in the production of gold nanoplates using algal extracts of *Chlorella vulgaris* (see below) [41].

**Figure 2:**
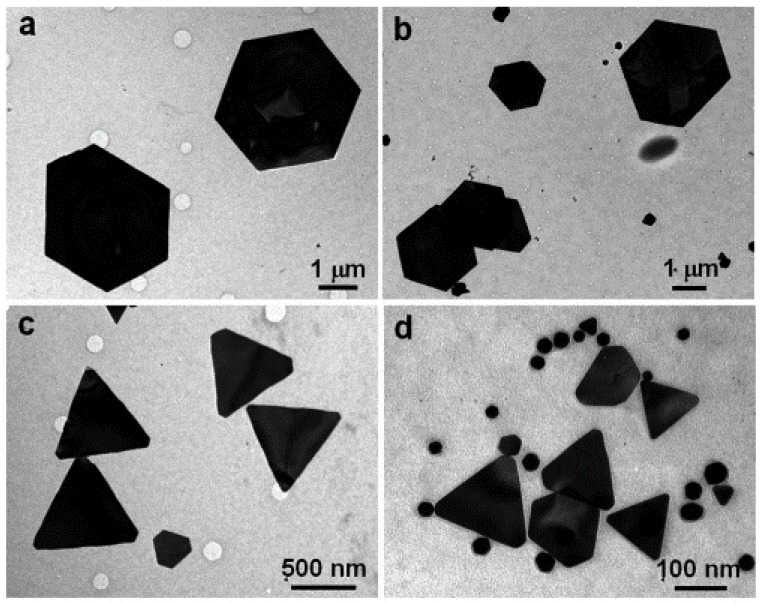
Representative TEM images of gold nanoparticles synthesized by the reduction of 5 mL of an aqueous solution of HAuCl_4_ at 10^−3^ M with (**a**) 0.2, (**b**) 0.3, (**c**) 0.5, and (**d**) 1.0 mL of lemon grass aqueous extract. Scale bars clockwise from top left are 1 µm, 1 µm, 500 nm and 100 nm. Reproduced from Reference [42]. Copyright (2005) American Chemical Society.

Playing with the initial amount of bioactive resources for the same content of Au(III), the aqueous extract of lemon grass, obtained by boiling finely cut leaves in water, was used to produce Au NP nanoplates of hexagonal, triangular and spherical shapes whose size may exceed hundreds of nm but of ~25 nm in thickness (Figure 2) [42]. A trend was obtained: when the amount of the extract is increased, the size diminishes and the shape evolves from exclusively hexagons to more spheres, triangles and truncated triangles forming the intermediate shapes. A previous work from the same team, using a 5-fold concentrated lemon grass broth, gave insights on the mechanistic formation of gold nanotriangles: first, a rapid reduction of Au(III) to Au(0) occurs, leading to the production of “liquid-like” spherical Au NPs which, subsequently, merge at room temperature to give birth to gold nanotriangles [43]. According to the authors of the study, the “liquid-like” character or “fluidity” of spherical Au NPs stems from the complexation of carbonyl groups, present in the lemon grass extract, to the surface of the as-generated Au NPs.

**Figure 3:**
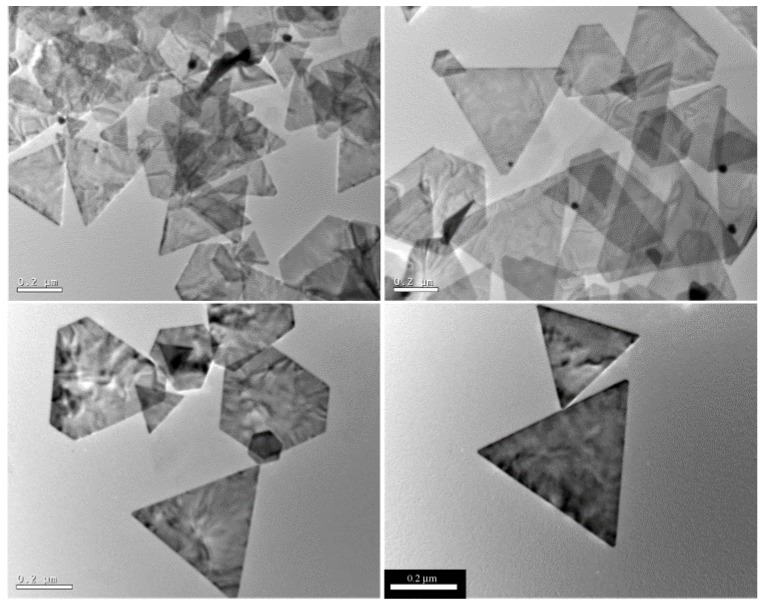
TEM images of gold nanoplates synthesized under the following experimental conditions: 5 mL of 1-day aged aqueous *Sargassum* extract + 5 mL of deionized H_2_O + 1 mL of 10 mM HAuCl_4_ aqueous solution, pH 7, room temperature for 3 h. All scale bars are 200 nm. Reproduced from Reference [44]. Copyright (2005) American Chemical Society.

Taking advantage of algal resources, the biosynthesis of Au NPs may lead to the production of nanoplates, nanorods or spheres. Gold nanoplates can be produced using extract from the brown seaweed *Sargassum* sp. [44] or from the green microalga *Chlorella vulgaris* [41]. In an experiment using *Sargassum* sp., Liu and coworkers powdered this brown macroalga to prepare the aqueous extract [44]. Then, the extract was mixed with an aqueous solution of HAuCl_4_ and several experimental parameters were screened, namely the pH, the reaction temperature, the age of the extract, the reaction time and the reactant concentrations—HAuCl_4_, the volume of the seaweed extract and the volume of added water. While a handful of the experimental sets did not give rise to any product, the others yielded a variety of results which can be grouped, i.e., different experimental sets generating similar products. To illustrate an example of the screened parameters, the Au NPs shown in Figure 3, composed mostly of triangular and truncated triangular nanoplates, resulted from mixing 5 mL of 1-day aged *Sargassum* extract, 5 mL of H_2_O and 1 mL of 10 mM HAuCl_4_, at room temperature and neutral pH for 3 h. Following another methodology, the same team managed to produce hexagonal, triangular and truncated triangular gold nanoplates using the extract of the unicellular green microalga *Chlorella vulgaris* [41]. To shed light on the underlying biochemical pathways, the same team isolated the protein governing the shape of the biogenic Au NPs—coined GSP for Gold Shape directing Protein. To do so, the lyophilized cells of *C. vulgaris* were dispersed in water and, two days later, the supernatant—the raw algal extract—was separated by filtration. Afterwards, the extract was serially fractionated using reverse phase high-performance liquid chromatography (RP-HPLC). The molecular weight of the implicated protein (GSP), present also in the raw algal extract, was estimated at 28 kDa. Interestingly, the GSP alone does not assure the production of gold nanoplates once challenged by Au(III). In fact, its content, determined by its optical density at 280 nm (OD_280_), and reaction time are key-parameters. It turned out that higher amounts of GSP and longer reaction times gave birth predominantly to gold nanoplates. Using atomic force microscopy (AFM), the thickness of gold nanoplates ranged from 9 nm to 17 nm. Importantly, the adsorption of the protein on the {111} planes of gold, which constitute the top and the bottom surfaces of the plates, directs the anisotropic growth of the crystals along the unprotected direction.

Parial et al. reported the production of gold nanorods using harvested, healthy cells of the cyanobacterium *Nostoc ellipsosporum* after they were exposed to an aqueous solution of HAuCl_4_ [45]. Furthermore, spherical Au NPs can be produced using, among others, an aqueous extract of the brown macroalga *Laminaria japonica* [46], an aqueous extract of the green macroalga *Prasiola crispa* [47] or living cells of several species of microalgae, maintained under their regular culturing conditions [48,49]. The latter is a one-step process that takes advantage of the enzymatic machinery present within the cells: the control over the shape and size of the Au NPs originates from the biocatalytic attributes (proteins) and the compartmentalized location of their intracellular synthesis. In other words, the reduction of Au(III) to Au(0) and, consequently, the formation of Au NPs occur within the lumen of the thylakoidal membranes leading, thus, to the appearance of spherical NPs with a narrow distribution in size. Afterwards, the NPs are released into the culture medium where they may form very stable colloids owing to the adsorption of cell-produced polysaccharides onto their surface. The colloidal stability may be impacted by culture media [50]. The procedure, based on using living cells of microalgae kept under their regular culturing conditions, is a promising route for the sustainable and scalable production of inorganic nanoparticles in fully automated photobioreactors [51,52]. Requirements for their design and amassed knowledge regarding the mechanistic aspects during the production of metallic NPs were reviewed recently [23]. Additionally, encapsulated cells of microalgae within inorganic [53] or combined inorganic/organic networks [54] constitute biohybrid platforms tested successfully in the production of Au NPs as the photosynthetic cells retain their bioreductive abilities.

### 2.2. Silver

Together with gold, silver is considered as a model metal to study the feasibility of NP synthesis using biological resources. Although tens of papers are released monthly, materials scientists have not given much attention to the screening of the experimental parameters in order to provide the as-produced Ag NPs with special features. For instance, Chandran and colleagues studied the effect of different amounts of the *Aloe vera* extract on the features of the as-produced Au NPs but they performed only one experiment with silver [40]. This sole experiment yielded spherical Ag NPs with a narrow distribution in size (15.2 ± 4.2 nm). Using a broth of dried leaves of *Cassia fistula*, Lin and coworkers reported the production of silver nanowires [55]. Reaction time proved to be the key-parameter that led to the production of such nano-structures. In fact, TEM images demonstrate clearly that, with increasing reaction time, spherical Ag NPs aggregate to give birth, at first, to nanorods, which keep growing to give rise, at last, to silver nanowires of 40–60 nm in diameter and more than 10 µm in length. Coffee extracts promote the production of very tiny spherical Ag NPs [39]. The biomass of the thermotolerant oleaginous microalga *Acutodesmus dimorphus*, used as the reducing agent, resulted in polydisperse spherical Ag NPs ranging from 2 nm to 15 nm [56]. Das et al. reported the synthesis of spherical Ag NPs, of 42–92 nm in size, using the supernatant of *Bacillus* strain CS 11 isolated from an industrial zone [57]. Dahoumane et al. described the intracellular biosynthesis of roughly spherical Ag NPs of 10–20 nm in diameter using living cells of the green microalga *Chlamydomonas reinhardtii* [58]. More recently, Shabnam et al. reported the biosynthesis of spherical Ag NPs of 5–30 nm in size using chloroplasts/thylakoids isolated from spinach via a light-driven process [59].

Xie et al. used the raw algal extract, made of the supernatant of lyophilized cells of *Chlorella vulgaris*, to carry out the biosynthesis of Ag NPs. As a result, 60% of the as-produced NPs were nanodisks of ~44 nm in size [60]. Through a slightly modified method described in another work of theirs [41], this group managed to direct the shape of Ag NPs by isolating, via a dialysis membrane, the proteins responsible for the preferential growth of the nanoparticles and the appearance of anisotropic nano-objects. As a consequence, low molecular weight proteins of less than 7 kDa were not able to promote the reduction of cationic silver whereas high molecular weight proteins did. Under these conditions, truncated triangular Ag nanoplates of ~48 nm in size represented ~80% of the observed NPs. Further physical and chemical treatments of the isolated bioactive molecules altered these features. For instance, when these biomolecules were heat-denatured, the percentage of truncated triangular plates dropped to ~60% and their size decreased to ~27 nm. Further experiments determined the key-roles played by the Tyr residue in the reduction process and the carboxylic groups of Asp in the shape determination of the as-fabricated Ag NPs.

**Figure 4:**
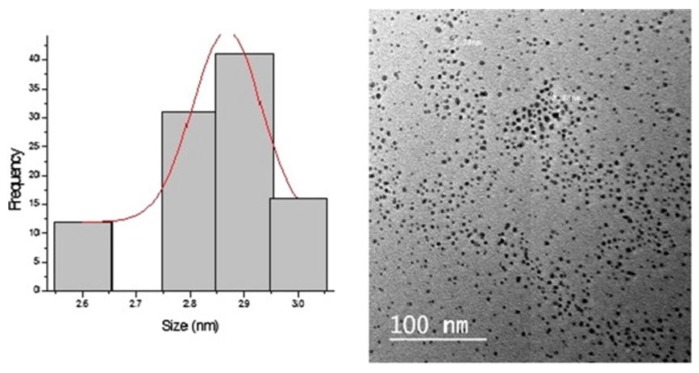
Size distribution histogram and HR-TEM micrograph of Ag NPs using the extract of the fungus *Rhizopus stolonifer* starting from an aqueous solution of AgNO_3_ at 10^−2^ M and at 40 °C. Reprinted from Reference [61]. Under a Creative Commons License. Copyright (2017) Elsevier.

To tightly control the size of the spherical nanoparticles produced using the aqueous extract of the fungus *Rhizopus stolonifer* starting from an aqueous solution of AgNO_3_, AbdelRahim et al. screened for the optimal experimental conditions by investigating the impact of cationic silver concentration and reaction temperature [61]. As displayed in Figure 4, 10^−2^ M of AgNO_3_ and 40 °C were found to be the optimal conditions for the synthesis of very tiny spherical Ag NPs with a narrow size distribution (2.86 ± 0.3 nm).

### 2.3. Palladium and Platinum NPs

The biosynthesis of palladium and platinum is less common in the scientific literature than that of gold and silver. However, a few papers report on the production of Pt NPs and Pd NPs of well controlled shapes and sizes. For instance, extracts of commercial coffee and tea promote the production of tiny spherical Pd NPs [39]. Likewise, the leaf extract of the plant *Delonix regia* proved efficient in the production of spherical Pd NPs of 2–4 nm in diameter and possessing interesting catalytic properties [62]. Similar results were obtained using other plant extracts [63,64]. Additionally, Omajali et al. described the intracellular synthesis of spherical Pd NPs by several strains of bacteria [65]. The size of these NPs appears to be strain- and electron donor-dependent. Moreover, Brayner and coworkers described the ability of cyanobacterial species to perform the intracellular biosynthesis of spherical Pd NPs and Pt NPs with a good control over their size [66].

The biosynthesis of tiny spherical Pt NPs of ~2.2 nm was carried out at neutral pH by mixing orange peel extract with hydrogen hexachloroplatinate (H_2_PtCl_6_) [67]. These NPs were found to be highly efficient in the catalyzed reduction of 4-nitrophenol to 4-aminophenol in the presence of NaBH_4_. On the other hand, square and rectangular Pt NPs were obtained by incubating a cell-free extract of the fungus *Fusarium oxysporum* in an aqueous solution of H_2_PtCl_6_ [68].

### 2.4. Metallic Alloys

The synthesis of alloy-based nanomaterials through biological routes is scarce. Thus, far, this literature reports the biosynthesis of alloy nanomaterials of noble metals among which silver and gold constitute the lion’s share. For instance, the biosynthesis of Ag-Au bimetallic alloy NPs is achieved using cyanobacteria [69,70], plant extracts [71,72], chloroplasts [73], an edible mushroom [35], fungi [74,75], and bacteria [76], while the biosynthesis of Pd-Au bimetallic alloy NPs is performed using bacteria [77,78]. Usually, the as-produced NPs are round-shaped but lack tight control over their size. Regarding the control over the composition, living cells of *Chlamydomonas reinhardtii* carried out the biosynthesis of very stable colloids made of spherical Ag-Au bimetallic alloy NPs whose compositions are well controlled and determined by the introduced ratios of [Ag^+^]/[Au^3+^] [58]. This was confirmed by the linear relationship between the SPR band and the ratio of Au(III). Moreover, it is possible to obtain Au@Pd core-shell nanoparticles using bayberry tannin via a one-step procedure [79] or *Cacumen platycladi* leaf extract via a two-step procedure [80]. In the first case, challenging aqueous mixtures of Au^3+^/Pd^2+^ of given compositions with an aqueous solution of bayberry tannin resulted in the appearance of core-shell NPs in which gold constitutes the core while palladium surrounds it by forming the shell [79]. Each experimental set yielded NPs possessing their own features. In the second example, the two-step procedure gave birth to flower-shaped Au@Pd core-shell NPs with a wide distribution in size [80].

## 3. Control of Size and Shape of Biologically Synthesized Oxide and Carbonate Nanoparticles

**Figure 5:**
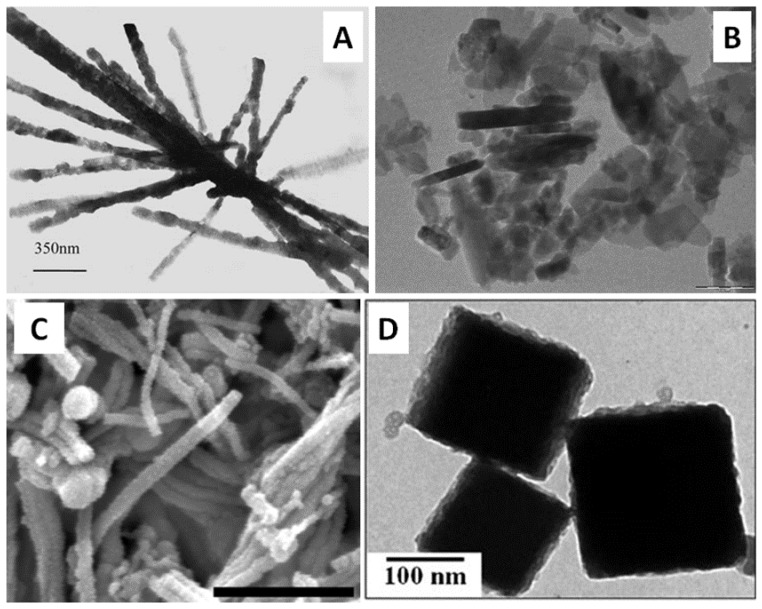
(**A**) CaCO_3_ nanorods synthesized by HEN-Qn1, a mixture of thermophilic bacteria strains. Adapted from Reference [86] with permission from Springer. (**B**) ZnO nanorods obtained by mixing biomass of seaweed *Padina tetrastomatica* and ZnCl_2_. Scale bar: 100 nm. Adapted from Reference [87] with permission from Wiley. (**C**) Hematite (Fe_2_O_3_) nanowires synthesized by biofilms of *Mariprofundus ferrooxydans* bacteria and annealed at 600 °C. Scale bar: 1 µm. Adapted from Reference [92]. Copyright (2016) American Chemical Society. (**D**) Nanocubes of magnetite (Fe_3_O_4_) produced by the fungus *Verticillium* sp. Adapted from Reference [93] with permission from Wiley.

There is a variety of oxide- and carbonate-based NP biosynthesis such as those made, for instance, of zinc oxide (ZnO) [81], copper oxide [82], magnetite (Fe_3_O_4_) [83], silica (SiO_2_) and titania (TiO_2_) [84], zirconia (ZrO_2_) [85], and calcium carbonate (CaCO_3_) [86]. In this latter example, CaCO_3_ nanorods were produced extracellularly and nanospheres intracellularly when a mixture of uncharacterized thermophilic bacterial strains, designated as HEN-Qn1, was incubated at 60 °C in the presence of an aqueous solution of CaCl_2_ (Figure 5A). This synthesis was made possible by the release of CO_2_ by the bacteria, as part of their aerobic metabolism. Nanorods of ZnO have been reported to be produced, under gentle heating, by mixing the biomass of the brown seaweed *Padina tetrastromatica*—ground dried macroalga—with aqueous solutions of three different precursors of zinc cations: ZnCl_2_, ZnSO_4_ and Zn(CH_3_COO)_2_ [87]. However, the process did not achieve any control over the size of the nanorods as their length varied from tens of nm to more than 100 nm (Figure 5B). The same team reported two years later, through a slightly modified procedure, the synthesis of ZnO NPs in the shape of rods, stars and plates depending on the Zn^2+^ precursor used [88]. Depending on the precursor, the resulted ZnO NPs may offer different shapes, such as nanoplates or star-like particles. The as-obtained ZnO NPs displayed both photocatalytic and bactericidal activities.

From a chemical composition point of view, the biosynthesis of iron oxide is more diverse compared to the previously mentioned oxides. This is related to the nature of the biomass used and on the species for a given microorganism. In the case of living cells of microalgae, cyanobacterium *Anabaena flos-aquae* and *Charophyta Klebsormidium flaccidum* promote the intracellular production of β-akageneite (β-FeOOH) nanorods [89,90], *Euglenozoa Euglena gracilis* accomplishes the intracellular synthesis of tiny nanoparticles of superparamagnetic 2-lines ferri-hydrite [91], and the biomass of the brown seaweed *Sargassum muticum* achieves the production of magnetite (Fe_3_O_4_) [83]. Besides the above mentioned nanorods of β-akageneite, iron oxide nanowires, made of hematite (Fe_2_O_3_), were fabricated by biofilms of *Mariprofundus ferrooxydans* bacteria [92]. The shape, size and morphology of these nanowires depend on the annealing temperature. For instance, the sample displayed in Figure 5C was obtained at 600 °C. Regarding their catalytic properties, the nanowires annealed at 800 °C proved to be the most efficient in the photocatalytic degradation of Rhodamine B under visible light. Additionally, nanocubes of iron oxides, composed predominantly of magnetite (Fe_3_O_4_), were generated by challenging a harvested biomass of the fungus *Verticillium* sp. with an aqueous solution of a mixture of Fe(II) and Fe(III) (Figure 5D) [93].

## 4. Control of Size and Shape of Biologically Synthesized Chalcogenide Nanoparticles

Chalcogenide nanoparticles are materials composed of a metal (Cd, Zn…) and a chalcogen (S, Se, Te). The physico-chemical methods for their synthesis are well established. For instance, cadmium sulfide (CdS) nanoparticles can be synthesized through the polyol process [94], a solvothermal route [95] or microwave irradiation [96]. Biological routes have proven efficient in the production of various sulfide-based nanomaterials, such as ZnS, CdS and PbS [97]. Through these eco-friendly routes, several microorganisms and extracts have been screened for their ability to promote the production of CdS NPs, such as bacteria [98], marine bacteria [99], photosynthetic bacteria [100], fungi [101,102], plant extracts [103,104,105], algae [106,107], pigments [108], and proteins [109], starting from the corresponding precursors of cadmium and sulfur. The as-produced CdS NPs can be exploited in photocatalytic applications [107] or valued for their biocidal properties [99]. These nano-objects may not have a well-defined shape and a controlled size [104], or be round-shaped with a good distribution in size [100]. Even though there is no control over the shape, the work by Dunleavy et al. demonstrates clearly, as shown in SEM (scanning electron microscopy) images, that the size of the synthesized CdS NPs using cystathionine γ-lyase increases with the reaction time [109]. This trend was corroborated by the fluorescence red-shift of these NPs.

The biosynthesis of quantum dots (QDs) of CdTe can be achieved using bacteria [110], yeast [111] and fungi [112]; of CdSe_0.5_S_0.5_ using bacteria [113]; and of CdSe using yeast [114]. It is possible to tailor the optical properties of CdSe QDs, produced by challenging the biomass of the yeast *Saccharomyces cerevisiae* by aqueous solutions of NaSe_2_O_2_ and CdCl_2_, through the screening of the following experimental parameters: time of addition, concentration and inoculating duration of NaSe_2_O_2_, and concentration and inoculating duration of CdCl_2_. For instance, by varying the latter—inoculating duration of CdCl_2_—from 14 h to 44 h, the color spans from green to red, yellow being the one of the sample obtained at the in-between inoculating duration.

**Figure 6:**
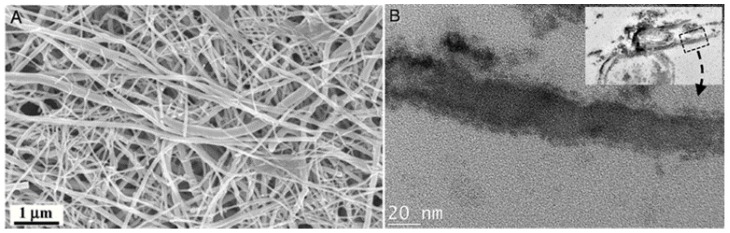
Electron micrographs of biogenic As-S nanotubes produced using the dissimilatory metal-reducing bacterium *Shewanella* sp. HN-41 once challenged by sources of arsenic and sulfur. (**A**) SEM image; (**B**) laterally sectioned TEM images. Scale bars from left to right are 1 µm and 20 nm. Adapted from Reference [115]. Copyright (2007) National Academy of Sciences.

Lee et al. described the biosynthesis of arsenic sulfide nanotubes using the dissimilatory metal-reducing bacterium *Shewanella* sp. HN-41 [115]. This was implemented by introducing Na_2_HAsO_4_, a source of As(V), and Na_2_S_2_O_3_, a source of S, to a culture of that microorganism grown under anaerobic conditions. This yielded nanotubes of As-S of several microns in length and tens of nanometers in width (Figure 6). X-ray diffraction (XRD) analyses revealed that the crystallinity of the As-S nanotubes improved with time. Moreover, their chemical composition evolved over time. While only orpiment (As_2_S_3_) was observed at day 9, several structures were recorded at day 50: orpiment, uzonite (As_4_S_5_), realgar (AsS), dimorphite (As_4_S_3_) and duranusite (As_4_S). This trend was confirmed using extended X-ray absorption fine structure (EXAFS).

Jiang and coworkers tested the possibility to extend the previous work to several strains of *Shewanella* [116]. Among the ten tested, only four strains proved able to promote the biosynthesis of As-S nanotubes once brought into contact with arsenate and thiosulfate. Following a different method, Mao et al. described the use of a genetically engineered virus, used as a biological template, for the surface nucleation of ZnS and CdS nanocrystals and their directed growth to form nanowires [117].

## 5. Conclusion and Perspectives

The present review summarizes critically selected literature data regarding the control of the shape, size and composition of inorganic nanomaterials synthesized via biological processes. These emerging and expanding bottom-up methodologies are based on the exploitation of the biomass of bacteria, fungi, algae and plants, in the form of aqueous extracts or whole cells. The nanomaterials generated belong to the chemical families of noble metals, carbonates, oxides and chalcogenides. By tuning the experimental parameters, such as the pH, the temperature, the reactant concentrations, e.g., amounts of the metal precursor and biomass extract, the reaction time, etc., it is possible to direct the shape of the biogenic nanomaterials, to control their size and diversify their chemical composition, and yield forms like, among others, nanospheres, nanoplates, nanorods and nanowires, possessing unique and valuable properties that can be exploited in numerous fields.

Nonetheless, a major gap in our current know-how resides in the small scale of NP production. Arguably, the majority of the protocols published and reviewed in this article are still little more than laboratory curiosities that have not been proven beyond the bench. It is, therefore, important to adapt the small-scale protocols to procedures that are amenable to standardized and robust scale-up in order to fulfill the promise of biogenic nanoparticles as a viable industrial activity. This constitutes a crucial current and future challenge for these emerging bioprocesses should be viable over the long term, be complementary to usual NP production routes or be competitive and embody an earnest alternative to them. The choice between plant- and microorganism-catalyzed processes has to be based on both economic and sustainability criteria. Because of plants’ wide availability, generally low cost and absence of toxicity, plus their extracts’ inherent richness in diverse forms of reducing and capping molecules, several lab-scale methodologies involving plant extracts should, in principle, be scalable for the production of nanomaterials with the desired characteristics. This observation remains applicable to macroalgae or seaweed, widespread in rivers, lakes and seas, and easy to harvest and handle. On the other hand, the industrial production of bacterial, fungal and, to a lesser extent, microalgal biomass is well established within the fermentation, brewery, food, pharmaceutical and many other sectors. Considerations such as the reaction time required—typically tens of hours or several days for microbes, including the culture and harvesting phases vs. minutes to hours for plant extracts, the temperature of the reaction—typically room temperature in plant extract-based methods vs. heating often required for the reaction or cultivation in microorganism-based methods, and, last but not least, the potential toxicity of some microorganisms vs. the generally nontoxic plant extracts seem to tilt the scale towards the side of plant and seaweed extracts [23,31]. There is, however, ample potential to scale up microbial-based methods as well [23,30]. For instance, it has been suggested that the fungal biosynthesis of Au NPs could be scaled up using bioreactors with immobilized biomass or incorporating the protein extracts immobilized in the form of thin films to minimize mass-transfer limitations and reaction time [30]. Additional consideration should be given to the cost-effective recovery of the Au NPs downstream from the bioreactor, hence extracellular production would be favored over intracellular one [30]. To get a complete panorama, several strains of microalgae carry out the process of noble metal NP biosynthesis intracellularly but, interestingly, the as-produced NPs are released into culture media to form stable colloids easing therefore their recovery [23].

The future of biogenic nanomaterials is very bright. Several science and engineering teams worldwide have been extending knowledge in the development of efficient and eco-friendly bioprocesses of synthesis and bio-application of nanomaterials, with desired shapes, controlled sizes and mastered compositions. To reach those goals with ever higher efficiency and predictability, materials scientists should bear in mind the importance of the combined screening for the right bioresources, i.e., bacteria, fungi, algae, plants and natural biomolecules, of the appropriate methodologies for the extraction/utilization of the active biomass and of the optimal experimental parameters.
